# Insomnia, psychiatric disorders and suicidal ideation in a National Representative Sample of active Canadian Forces members

**DOI:** 10.1186/s12888-017-1372-5

**Published:** 2017-06-06

**Authors:** J. D. Richardson, A. Thompson, L. King, B. Corbett, P. Shnaider, K. St. Cyr, C. Nelson, J. Sareen, J. Elhai, M. Zamorski

**Affiliations:** 1 Western University, 1151 Richmond St, London, ON N6A 3K7 Canada; 2 McMaster University, 1280 Main St W, Hamilton, ON L8S 4L8 Canada; 3 Parkwood Operational Stress Injury Clinic-Parkwood Institute-St. Joseph’s Health Care London, 550 Wellington Rd, London, ON N6C 0A7 Canada; 4 Canadian Forces Health Services Group and Department of Family Medicine, University of Ottawa, 75 Laurier Ave E, Ottawa, ON K1N 6N5 Canada; 5 Stamford International University, Prawet, Bangkok, 10250 Thailand; 6 St. Joseph’s Healthcare Hamilton, 2757 King Street East, Hamilton, ON L8G 5E4 Canada; 7 Ryerson University, 350 Victoria St, Toronto, ON M5B 2K3 Canada; 8 University of Manitoba, 66 Chancellors Cir, Winnipeg, MB R3T 2N2 Canada; 9 Deer Lodge Centre Operational Stress Injury Clinic, 2109 Portage Avenue, Winnipeg, MB R3J 0L3 Canada; 10 University of Toledo, 2801 W Bancroft St, Toledo, OH 43606 USA

**Keywords:** Sleep, Suicidal ideation, Military, Mental health, Depression, Posttraumatic stress disorder, Insomnia

## Abstract

**Background:**

Past research on the association between insomnia and suicidal ideation (SI) has produced mixed findings. The current study explored the relationship between insomnia, SI, and past-year mental health status among a large Canadian Forces (CF) sample.

**Method:**

Data was obtained from the 2013 Canadian Forces Mental Health Survey (CFMHS), and included a large representative sample of Canadian Regular Forces personnel (*N* = 6700). A series of univariate logistic regressions were conducted to test individual associations between past-year mental health status, insomnia, and potential confounds and SI. Mental health status included three groups: 0, 1, or two or more probable diagnoses of posttraumatic stress disorder (PTSD), major depressive disorder (MDD), generalized anxiety disorder (GAD), panic disorder (PD) and alcohol abuse/dependence. Stepwise multivariate logistic regression was used to assess the relationship between insomnia and SI with mental health status as a moderator.

**Results:**

40.8% of respondents reported experiencing insomnia. Both insomnia and number of mental health conditions incrementally increased the risk of SI. However, past-year mental health status was a significant moderator of this relationship, such that for CF personnel with either no (AOR = 1.61, 1.37–1.89) or only one past-year mental health condition (AOR = 1.39, 1.12–1.73), an incremental increase in insomnia was associated with an increased likelihood of SI. However, in personnel with two or more past-year mental health disorders, insomnia was no longer significantly associated with SI (AOR = 1.04, 0.81–1.33).

**Conclusions:**

Insomnia significantly increased the odds of SI, but only among individuals with no or one mental health condition. Findings highlight the importance of assessing insomnia among CF members in order to further suicide prevention efforts.

## Background

The past decade of armed conflict in Afghanistan and Iraq has been associated with increased rates of suicidal behaviour, including suicidal ideation, plans, and attempts, in US military personnel. [1–6] Findings from other nations have been less dramatic—perhaps related to smaller military populations hence greater difficulty detecting significant trends for rare events like suicide—but Canada, at least, may now be seeing similar patterns. [7] Several studies have pointed to a greater risk of suicidality in modern veterans—a finding not detected in veterans of earlier conflicts. [7–9]

The role of combat-related psychiatric disorders in rising military suicide rates has not been clear-cut. [10] Although conditions which can emanate from trauma exposure, such as combat-related Posttraumatic Stress Disorder (PTSD), [11] Major Depressive Disorder (MDD) [12–15] and various anxiety disorders, [16–18] each independently predict suicidal behaviour, several studies have shown little or no significant relationship between deployment and completed suicide. [2] Therefore, it is important to examine other potential drivers of suicidal behaviour.

Insomnia is a prevalent problem among military cohorts both during and following deployment [19, 20], and has been linked to suicidal ideation (SI), attempts, and completed suicide in both military and civilian populations [2, 21–30]. Data obtained from the Deployment Life Study [31] demonstrated that nearly half of their sample (48.6%) exceeded the clinically significant threshold for sleep problems [32]. There are also a number of compelling reasons why insomnia may contribute to suicidal behaviour [33]. For example, Bernert and Joiner report that poor sleep quality, via frightening dream content and insomnia, can disrupt within-sleep mood regulation processes. This in turn can impart a negative and potentially long-lasting effect on psychopathology, and may influence the association between sleep and suicidality [23]. Insomnia has also been shown to desensitise the serotonergic (1A) receptor system in rats [34], and a number of studies have linked serotonergic deregulation with past suicidal attempts, future suicide prediction, and completed suicide [23]. Thus, insomnia may play a prominent role in the formation of suicidal behaviour, and presents as a potential target for interventions aimed at preventing suicide.

A challenge in understanding the association between insomnia and suicidal behaviour is that sleep problems are core symptoms of common psychiatric disorders including MDD, PTSD, and Generalized Anxiety Disorder (GAD) [35, 36]. Thus, it remains unclear whether the effect of insomnia on suicidal behaviours is better accounted for by the presence of psychiatric conditions that may include insomnia as a marker of symptom severity. Consequentially, it is important to examine the independent influence insomnia confers onto risk of suicidal behaviours by controlling for the psychiatric conditions.

Studies controlling for the presence of psychiatric conditions while examining the association between insomnia and risk of suicidal behaviours have yielded mixed results. One potential explanation for discrepant findings is that the relationship between insomnia and suicidality is moderated by mental health problems. Accordingly, the effect of insomnia on suicidality may differ among those with, compared to those without, a mental disorder.

While past studies have statistically controlled for the effect of psychopathology, no studies, especially in military samples, have examined the interactive link between insomnia and mental health status in predicting suicidal ideation. Better understanding such effect modification may help explain the discrepant findings and aid in identifying individuals for which sleep-oriented interventions might attenuate risk of suicidal behaviours. For example, Bernert and colleagues [22] and Liu [37] noted that although experiencing nightmares was significantly related to elevated suicidal symptoms, the relationship between insomnia and suicidality failed to reach significance. In a treatment-seeking sample of predominantly veterans, Richardson and colleagues [38] failed to find significant associations between either insomnia or nightmares and SI when controlling for probable diagnoses of PTSD, MDD, GAD, or alcohol use disorder (AUD). Instead, MDD emerged as the only significant predictor of SI. Similarly, Bryan and colleagues^43^ found that insomnia severity was not directly associated with concurrent or prospective suicidal ideation in three military samples when adjusting for depression.

Conversely, others have found an association between symptoms of insomnia and suicidal behaviour that remained significant after controlling for common psychiatric conditions [39–41]. Specific to military populations, Ribeiro and colleagues [25] found that insomnia symptoms predicted SI even after controlling for depression, hopelessness, PTSD, anxiety, and alcohol abuse. Pigeon and colleagues [42] also demonstrated that veterans’ time to completed suicide was associated with sleep disturbances after adjusting for mental health, age, and substance-use symptoms.

Given a recent increase in reported rates of suicide among military members, [1–6, 43] coupled with the few studies on insomnia and suicide in military members as opposed to other populations currently available, the authors of the current study hypothesize that insomnia does contribute to SI amongst military personnel, and that an interactive effect between insomnia and mental health conditions such as PTSD may exist.. The current paper used data from a 2013 population-based mental health survey of serving Canadian military personnel to explore: 1) The contribution of insomnia towards SI while controlling for common mental disorders; and 2) The possibility of interactions between insomnia and mental disorders with respect to SI.

## Methods

### Participants and procedure

Data for this study were obtained from the 2013 Canadian Forces Mental Health Survey (CFMHS). This survey aimed to assess the mental health status and service use of currently serving Regular Force and Reserve Force personnel, as well as the impacts of any perceived needs; and to aid in the identification of occupational and non-occupational risk and resilience factors. Using an administrative list from the Canadian Forces Human Resource Management System, CF Regular and Reserve Force members were stratified by rank, CF environment (e.g., Army, Navy, and Air), base, gender, and language. Systematic sampling was then used to ensure a nationally-representative sample of the CF. A total of 8393 Regular Force and 1867 Reserve Force members were invited to participate in the survey, which garnered response rates of 79.8% and 78.7%, respectively. Data were collected by trained Statistics Canada employees using computer-assisted personal interviews, and participation in the survey was voluntary. Further information about the survey can be found at http://www23.statcan.gc.ca/imdb/p2SV.pl?Function=getSurvey&SDDS=5084. For the analyses of the current study, only Regular Force personnel data were included (*N* = 6700).

### Measures

#### Socio-demographic and military characteristics

Socio-demographic and military covariates measured were sex, age cohort (17–29, 30–39, 40–49, 50–60 years), race (white, non-white), marital status (married/common law, divorced/separated/widowed, single/never married), language (English, French), and deployment to Afghanistan (yes, no).

#### Insomnia

A single item was used to assess insomnia. In the General Health section of the survey, which examined current perceived health status and social well-being, military personnel were asked, "how often do you have trouble going to sleep or staying asleep?" Responses were provided using a 5-point Likert-scale (1 = “None of the time,” 2 = “A little of the time,” 3 = “Some of the time,” 4 = “Most of the time,” and 5 = “All of the time”). In the present study, insomnia was assessed as a continuous variable in order to examine whether the odds of suicidal ideation changed with increases in the severity of insomnia. The decision to examine continuous changes in insomnia as opposed to categories of insomnia was based on the small number of respondents who indicated experiencing insomnia “most of the time” and “all of the time”. Furthermore, the artificial categorization of a continuous predictor can lead to a substantial loss of power in tests of effect modification.

#### Mental health status

The presence or absence of meeting the criteria for probable PTSD, MDD, GAD, AUD, and panic disorder (PD) in the past 12 months was measured. Past-year mental health disorders were assessed using The World Health Organization Composite International Diagnostic Interview (WHO-CIDI) according to DSM-IV criteria [35]. The WHO-CIDI is a lay-administered instrument shown to have acceptable concordance with clinical diagnostic instruments [44, 45]. In the present study, CF personnel were classified into one of three mental health status groups: 1) No past-year mental health disorder; 2) One past-year mental health disorder; or 3) Two or more past-year mental health disorders.

#### Past-year suicidal ideation

A single item measured, yes or no, whether respondents had seriously thought about committing suicide or taking their own life in the past 12 months. This question has been used as an indicator of past-year SI in previous military research [11, 46, 47].

### Statistical analyses

Analyses were conducted using SAS 9.2 and STATA 13.0. [48] Final survey data were weighted and provided by Statistics Canada to reflect the initial sampling weight and account for non-response and the removal of any out-of-scope units. To account for the complex design of the 2013 CFMHS, variance estimates were calculated using the 500 weighted bootstrap samples generated by Statistics Canada. According to Statistics Canada’s confidentiality guidelines for this survey, only weighted estimates were reported and weighted sample estimates were rounded to the nearest 20. As outlined in the 2013 CFMHS master documentation provided by Statistics Canada, estimates with a coefficient of variation between 16.6% and 33.3% were flagged (E) as potentially unreliable while estimates with a coefficient of variation greater than 33.3% were flagged (F) and suppressed as unreliable. Caution should be used in interpreting findings associated with estimates flagged (E) potentially unreliable as these estimates are derived from subsamples with high sampling variability.

Preliminary analyses using a series of univariate logistic regression models were conducted to test the individual association of past-year mental health and mental health status, insomnia, and the socio-demographic and military covariates with past-year SI. Odds ratio estimates with 95% confidence limits were used to interpret significant differences in the odds of reporting past-year SI.

**Fig. 1:**
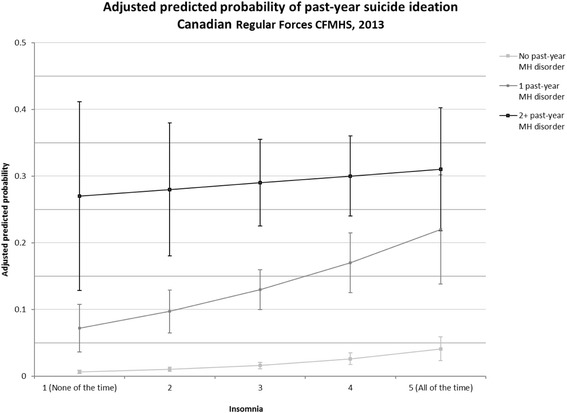
Richardson et al. Adjusted predicted probabilities estimating the risk of past-year suicide ideation associated with the effects of the number of past-year MH disorders at different levels of insomnia

Following these preliminary analyses, stepwise multivariate logistic regression was used to test effect modification. In the first step, past-year SI was regressed on past-year mental health status and insomnia adjusting for significant covariates. In the second step, an interaction between past-year mental health status and insomnia was added as a predictor of past-year SI. To interpret the interaction, adjusted simple odds ratios with 95% confidence intervals were tested to estimate the change in odds of past-year SI associated with increases in insomnia in each mental health status group. The adjusted ratio of odds ratios (AROR) and 95% confidence intervals were tested to assess significant differences in the odds of past-year SI associated with insomnia between past-year mental health status groups. Furthermore, as depicted in Fig. 1, average adjusted predicted probabilities with 95% confidence intervals were used to estimate the probability of past-year SI at different levels of insomnia in each mental health status group.

## Results

### Sample description

Table 1 depicts the socio-demographic and military characteristics and prevalence rates of past-year mental health disorders and status, insomnia, and past-year SI in the 2013 regular CF population. Personnel were predominantly white, English-speaking males with an average age of approximately 35 years and were mostly either married or living common-law. Nearly half (45.1%) had been deployed at least once to Afghanistan. The most common past-year mental health disorder was MDD (8.0%) followed by PTSD (5.3%), GAD (4.7%), AUD (4.5%), and PD (3.4%). Considering the past-year mental health status of personnel, 84.0% reported no mental health disorder, 9.9% reported one mental health disorder, and 6.1% reported two or more mental health disorders. Of the respondents reporting two or more mental health disorders, PTSD was reported by 57.6% of respondents, MDD was reported by 75.4% of respondents, GAD was reported by 57.6% of respondents, AUD was reported by 26.2% of respondents while PD was reported by 39.3% of respondents. Less than half of personnel (40.8%) reported experiencing insomnia some, most, or all of the time with 5.3% of personnel experiencing insomnia all of the time; whereas 30.45% of personnel reported no difficulty sleeping. Finally, 4.3% of personnel reported past-year SI.

**Table 1:** Weighted sample and prevalence estimates representative of 2013 Regular Canadian Forces personnel

		Proportion %	Weighted count
Gender	Male	86.1	55,480
	Female	13.9	8920
Age cohort	17–29 years	32.9	21,200
	30–39 years	32.3	20,820
	40–49 years	25.5	16,440
	50 years and older	9.2	5940
Marital status	Married, Common-law	65.6	42,200
	Separated, Divorced, Widowed	7.5	4840
	Single, Never married	26.9	17,300
Language	English	78.58	50,600
	French	21.42	13,800
Race	White	93.7	60,180
	Non-white	6.3	4080
Deployment	Afghanistan at least once	45.1	29,060
Insomnia	None of the time	30.5	19,600
	A little of the time	28.7	18,460
	Some of the time	24.4	15,720
	Most of the time	11.1	7140
	All of the time	5.3	3440
12 month prevalence rate	MDD	8.0	5120
	PTSD	5.3	3340
	GAD	4.7	3000
	Alcohol Abuse and Dependency	4.5	2880
	PD	3.4	2140
	Suicide ideation	4.3	2740
Past-year mental health status	No disorder	84.0	52,120
	1 disorder	9.9	6120
	2 or more disorders	6.1	3820

### Effect modification

As depicted in Table 2, the results of the preliminary univariate logistic analyses supported the subsequent examination of past-year mental health status categorizing respondents based on the presence of any past-year mental health disorder. Each past-year mental health disorder including MDD, PTSD, GAD, PD, and AUD was individually associated with past-year SI. Following, the odds of reporting past-year SI was significantly different for each past-year mental health disorder group when compared to the “no past-year mental health disorder” group. Furthermore, insomnia was significantly associated with past-year SI. Finally, marital status was the only significant covariate associated with past-year SI and was subsequently adjusted for in the test of effect modification.

**Table 2:** Prevalence of past-year suicidal ideation and univariate associations of past-year suicidal ideation with past-year mental health disorders and status, insomnia, and socio-demographic and military characteristics

	Reported past-year suicide ideation		Univariate association with past-year suicide ideation
	Weighted count	Row %	Unadjusted OR (95% CI)
PTSD			
No^(Ref)^	1720	2.9	1.00
Yes	940	27.9	13.06 (9.47–18.00)**
MDD			
No^(Ref)^	1340	2.2	1.00
Yes	1400	27.5	16.44 (12.18–22.21)**
GAD			
No^(Ref)^	1900	3.1	1.00
Yes	820	27.3	11.65 (8.35–16.26)**
PD			
No^(Ref)^	2180	3.6	1.00
Yes	540	24.9	8.98 (6.21–12.98)**
Alcohol Abuse and Dependency			
No^(Ref)^	2300	3.7	1.00
Yes	440	15.1	4.55 (3.04–6.81)**
Insomnia			
None of the time	200^(E)^	1.0	
A little of the time	400^(E)^	2.2	
Some of the time	860	5.5	
Most of the time	780	10.9	
All of the time	500	14.7	
Incremental continuous change			2.05 (1.85–2.28)**
Past-year mental health status			
No mental health disorder^(Ref)^	640	1.2	1.00
1 mental health disorder	820	13.4	12.23 (8.33–17.96)**
2 or more mental health disorders	1140	29.8	33.71 (22.82–49.79)**
Sex			
Male^(Ref)^	2380	4.3	1.00
Female	380^(E)^	4.3	0.98 (0.65–1.48)^(E)^
Age			
17–29^(Ref)^	1340	2.2	1.00
30–39	1060	5.1	1.22 (0.86–1.74)
40–49	600	3.6	0.87 (0.59–1.28)
50–60	200^(E)^	3.4	0.77 (0.47–1.25)^(E)^
Race			
White^(Ref)^	2580	4.3	1.00
Non white	160^(E)^	3.9	0.91 (0.54–1.53)^(E)^
Marital status			
Married, Common-law^(Ref)^	1560	3.7	1.00
Divorced, Separated, Widowed	340^(E)^	7.1	1.94 (1.31–2.89)^(E)^**
Single, Never married	840	4.9	1.34 (0.96–1.86)
Language			
English^(Ref)^	2300	4.5	1.00
French	440^(E)^	3.2	0.70 (0.48–1.02)^(E)^
Deployment to AFG			
Yes^(Ref)^	1340	4.6	1.00
No	1400	4.0	1.16 (0.89–1.51)

^(Ref)^ Reference category for categorical predictors

^E^Use with caution

*Significantly different from reference at *p* < .01

**Significantly different from reference at *p* < .001

Table 3 presents the results of the test of effect modification. In the first model, both past-year mental health status and insomnia were independently associated with past-year SI. In comparison to personnel with no past-year mental health disorder, the odds of reporting SI were approximately 10 times greater in personnel with one past-year mental health disorder and approximately 22 times greater in personnel with 2 or more past-year mental health disorders. Findings also demonstrated that the odds of experiencing past-year SI increased 1.34 times with each increase in the level of insomnia reported. The results of the second model revealed a significant interaction between past-year mental health status and insomnia associated with past-year SI. There was a significant increase in the odds of past-year SI associated with each increment in insomnia in personnel reporting no past-year mental health disorder (AOR = 1.61, 1.37–1.89) and one past-year mental health disorder (AOR = 1.39, 1.12–1.73). In contrast, incremental changes in insomnia did not significantly relate to changes in the odds of past-year SI (AOR = 1.04, 0.81–1.33) in personnel with two or more mental health disorders.

**Table 3:** Stepwise multivariate logistic regression model examining past-year mental health status as an effect modifier of the association between insomnia and past-year suicide ideation

	Adjusted OR (95% CI)
Model 1	
Insomnia	1.34 (1.18–1.52)**
Past-year mental health status	
No mental health disorder ^(Ref)^	1.00
1 mental health disorder	9.82 (6.55–14.71)**
2 or more mental health disorders	22.40 (14.23–35.25)**
Model 2	
Insomnia*past-year mental health status	
	Adjusted OR (95% CI)
Insomnia x No mental health disorder	1.61 (1.37–1.89)*
Insomnia × 1 mental health disorder	1.39 (1.12–1.73)*
Insomnia × 2 or more mental health disorders	1.04 (0.81–1.33)
	Adjusted ratio of OR (95% CI)
Insomnia × 1 mental disorder vs Insomnia x No mental health disorder	1.16 (0.88–1.52)
Insomnia × 2 mental disorder vs Insomnia x No mental health disorder	1.55 (1.15–2.07)*
Insomnia × 2 mental disorder vs Insomnia × 1 mental health disorder	1.34 (0.97–1.85)

Model 1: Adjusted logistic regressions adjusted for marital status with sleep disturbances and past-year mental health status entered in the same model

Model 2: Same variables as model 1 with the addition of the interaction between sleep disturbances and past-year mental health status

^(Ref)^ Reference category

*Significantly different from reference at *p* < .01

**Significantly different from reference at *p* < .00

Figure 1 presents the average adjusted predicted probabilities depicting the differences in the odds of past-year SI associated with insomnia between mental health status groups. The rate of increase in the odds of past-year SI associated with increments in insomnia was not significantly different between personnel reporting no past-year mental health disorder and one past-year mental health disorder (AROR = 1.16, 0.88–1.52, *p* > .05). As shown in Fig. 1, the probability of past-year SI associated with the report of insomnia from “none of the time” to “all of the time” increased from less than 1 % to 4.1 in personnel with no past-year mental health disorder and increased from 7.2% to 22% in personnel with one past-year mental health disorder. In contrast, the rate of increase in the odds of past-year SI associated with incremental changes in insomnia was significantly greater, approximately 1.5 times, in personnel reporting no past-year mental health disorder in comparison to personnel reporting two or more past-year mental health disorders (AROR = 1.55, 1.15–2.07, *p* < .005). The rate of increase in the odds of past-year SI associated with incremental changes in insomnia was not found to be significant in personnel reporting one past-year mental health disorder in comparison to personnel reporting two or more past-year mental health disorders (AROR = 1.34, 0.97–1.85, *p* = .08). As shown in Fig. 1, the probability of past-year SI remained relatively stable across levels of insomnia at 27% at “none of the time” and 31% at “all of the time” in personnel with two or more past-year mental health disorders.

## Discussion

Consistent with previous research in military samples, results indicated that insomnia is commonly reported among service members. [19, 20] As expected, from the univariate models the psychiatric disorders (PTSD, MDD, GAD, PD and AUD), as well as insomnia, each predicted SI on their own. However, these models do not account for the overlapping variance that is shared between disorders. Further, insomnia cumulatively increased the odds of SI, such that insomnia severity was associated with an increased likelihood of reporting of SI.

A number of novel findings emerged regarding number of mental health diagnoses. First, a cumulative effect was demonstrated such that the odds of experiencing SI increased with additional mental health diagnoses, indicating the potential for a dose-response relationship between mental illness and risk of SI. Mental health diagnosis also conferred a moderating effect such that insomnia incrementally increased the odds of SI, but only among those with no or one mental health diagnosis present. Conversely, no significant cumulative effect emerged between insomnia and SI for respondents with two or more mental health diagnoses. The findings suggest there may be a ceiling effect on the relative impact of insomnia on suicidal ideation above and beyond the effect of comorbid mental health disorders. Therefore, when comorbidity is present, the influence of insomnia on SI is minimized due to already-existing mental health symptoms affecting the formation and maintenance of suicidal behaviour. Alternatively, it may be that when the frequency and/or severity of mental health symptoms is relatively low, incremental increases in sleep disturbances may be perceived as increasingly pervasive and burdensome to well-being.

The current study found that approximately 41% of the sample reported experiencing insomnia some, most, or all of the time. This approximates the rate of sleep problems reported in the Deployment Life Study (48.6%) [32], but is noticeably higher than the reported rates of insomnia (16–21%, as measured by frequency) in several general population samples, according to an epidemiologic review by Ohayon [49] and marginally higher than the rates averaged across adult general populations (30–36%) by Morin and Jarrin [50]. These findings suggest that military personnel may be at increased risk of insomnia compared to their civilian counterparts, and may benefit from additional screening and intervention targeting insomnia. Additionally, the current study found that approximately 10% of the sample met screening criteria for one mental health disorder and an additional 6% met screening criteria for two or more. While estimates of mental health disorder prevalence among military personnel range widely, this finding is similar to that of Hoge and colleagues, which found that 11.3% of military personnel deployed to Afghanistan as part of Operation Enduring Freedom screened positively for a mental health disorder [51]. These rates are lower than that of the American general population (26.2% for any past-year DSM-IV disorder,) as reported by Kessler and colleagues [52]. This discrepancy may be, at least in part, attributable to the “healthy soldier” effect. Finally, the current study found that 4.3% of participants reported past-year SI. A nationally-representative study of the Canadian general population found a past-year SI rate of approximately 3.3% [53], while data from the US military’s Post-Deployment Health Assessment survey found that 1.3% of participants reported “some” to “a lot” of SI [51]. Similarly to mental health disorders, SI may be susceptible to underreporting in actively serving military populations for a number of reasons (“healthy soldier” effect, fears about job security, stigma, etc.). The incongruity between the rate reported in the current study and Hoge and colleagues is interesting; further research pertaining to SI and its management among national armies may help elucidate important differences affecting these rates.

The current study gives rise to important clinical implications. Foremost, sleep-oriented interventions may attenuate risk of suicidal behaviours, particularly amongst military personnel experiencing insomnia independently of mental health comorbidities. For those with mental health conditions, studies documenting the persistence of insomnia despite treatment for conditions such as MDD [54] and PTSD [55] suggest sleep problems that co-occur with psychiatric conditions may not resolve themselves as a result of treatments targeting specific mental health diagnoses. Thus, treatments adapted to complement other interventions are advisable. For example, the following may help to reduce risk of SI: inclusion of psychoeducation related to sleep disturbances and PTSD, psychotherapy to target nightmares, such as nightmare rescripting or imagery rehearsal therapy [56, 57], cognitive-behavioural therapy for insomnia [58], use of pharmacotherapy such as prazosin for trauma related nightmares [59–61] or risperidone [62], or low-dose sedating antidepressants or hypnotics [63–65]. These treatments may provide additional gains and increased quality of life among individuals struggling with sleep problems and mental disorders.

Although the current study has several strengths, including the use of a nationally- representative Canadian military sample that includes individuals deployed to the recent conflict in Afghanistan, its limitations are worth noting. Although “trouble going to sleep or staying asleep” was part of questionnaire inferred to assess current health problems, no time frame was specified. Thus, we cannot guarantee that mental health conditions and insomnia occurred at the same time participants experienced SI. We also used single items to measure suicidality and insomnia. While not ideal, a similar single-item measures have been used elsewhere to study insomnia and suicide [38]; however, the use of more elaborate measures of insomnia and SI in future studies may further clarify the relationship between insomnia, mental health conditions, and SI identified in the current study. Additionally, the item used to measure insomnia was worded to include both problems falling or staying asleep as a single item. It is possible that differences exist between those who endorse difficulty falling asleep vs. those who experience problems staying asleep, and that individuals who report difficulty both falling and staying asleep differ from those who experience difficulty either falling *or* staying asleep.

It is also worthwhile noting that SI is distinct from other suicidal behavior, such as engaging in self-harm, and suicide planning or attempts. Importantly, while the rate of SI in the current study was higher than that of a similar US military population, this does not necessarily mean Canadian soldiers are at a greater risk of suicidal behaviors than their American counterparts. Further studies assessing a range of suicidal behaviors are recommended in order to better understand the implications of this finding. We were also unable to examine the effect of specific mental health conditions (i.e., PTSD, MDD, GAD, PD and AUD) due to a limited number of respondents reporting each disorder. Further, the large number of mental disorders we assessed made it impractical to test individually for single-disorder interactions. The cross-sectional design also limited our ability to evaluate causal relationships between insomnia, psychiatric conditions, and SI. Further, the current findings are limited to actively serving military members, and cannot be generalized to other psychiatric or veteran populations. Lastly, the self-report nature of the data means we must consider the possibility of underreporting symptoms of mental health conditions and SI, given the nature of the study population.

Further research regarding the effectiveness of sleep-related treatments to reduce SI in military members is certainly warranted. Longitudinal research may provide insight into potential causal pathways between variables examined in the current study, both in the development of these conditions and their treatment. Given the low base rates of suicide completions and attempts, future studies should also consider collecting data from a sample in which these rates are higher in order to evaluate whether the same factors that contribute to increased risk for SI also contributes to increased risk for suicide completions and attempts, such as the Army Study to Assess Risk and Resilience in Service members (Army STARRS) studies [5, 66]. Although the current study examined the risk of mental-health diagnosis categorically, further research will benefit from investigating whether certain diagnoses confer greater risk or burden than others. Finally, research targeting military clinical samples with specific psychiatric disorders is needed to test whether the independent risk of SI associated with insomnia is consistent when controlling for the influence of each mental health disorder separately.

## Conclusions

Military suicide remains an important issue critically requiring effective prevention strategies. We found a high prevalence of insomnia in a general military population, and that mental health status moderated the relationship between sleep disturbances and SI, such that insomnia was associated with SI among CAF personnel without mental health diagnoses, or only one mental health disorder, but not among those with comorbid mental health disorders. Findings highlight the potential benefits of assessing sleep disturbances in CAF personnel in order to further suicide prevention efforts, and inform preventative interventions targeting sleep in both clinical and non-clinical populations.

## Abbreviations

AOR: Adjusted odds ratio
AROR: Adjusted ratio of odds ratio
AUD: Alcohol use disorder
CBT-I: Cognitive behavioural therapy for insomnia
CF: Canadian Forces
CFMHS: Canadian Forces Mental Health Survey
DSM-IV: Diagnostic and Statistical Manual of the American Psychiatric Association, Fourth Edition
GAD: Generalized Anxiety Disorder
MDD: Major Depressive Disorder
PD: Panic disorder
PTSD: Posttraumatic Stress Disorder
RegF: Regular Force
SI: Suicidal ideation
WHO-CIDI: World Health Organization Composite International Diagnostic Interview

## References

[1] Harrell MC, Berglass N: Losing the battle: the challenge of military suicide. Policy brief. In. Edited by Security CfaNA. Washington, DC; 2011

[2] Nock MK, Deming CA, Fullerton CS, Gilman SE, Goldenberg M, Kessler RC, McCarroll JE, McLaughlin KA, Peterson C, Schoenbaum M (2013). Suicide among soldiers: a review of psychosocial risk and protective factors. Psychiatry.

[3] Ursano RJ (2013). Suicide: a national health challenge, an army health threat. Psychiatry.

[4] Thompson JM, Zamorski MA, Sweet J, VanTil L, Sareen J, Pietrzak RH, Hopman WH, MacLean MB, Pedlar D (2014). Roles of physical and mental health in suicidal ideation in Canadian Armed Forces regular Force veterans. Can J Public Health.

[5] Ursano RJ, Heeringa SG, Stein MB, Jain S, Raman R, Sun X, Chiu WT, Colpe LJ, Fullerton CS, Gilman SE et al: Prevalence and Correlates of Suicidal Behavior among New Soldiers in the U.S. Army: Results from the Army Study to Assess Risk and Resilience in Servicemembers (Army Starrs). Depress Anxiety 2015, 32(1). 10.1002/da.22317 PMC5113817 25338964

[6] McCarthy M (2014). Suicide rates double among US soldiers between 2004 and 2009, research shows. BMJ.

[7] Rolland-Harris E, Whitehead J, Matheson H, Zamorski MA: 2015 report on suicide mortality in the Canadian Armed Forces (1995 to 2014). In. Edited by Defense DoN. Ottawa: Department of National Defense; 2015.

[8] Kaplan MS, Huguet N, McFarland BH, Newsom JT (2007). Suicide among male veterans: a prospective population-based study. J Epidemiol Community Health.

[9] Zamorski MA (2011). Suicide prevention in military organizations. Int rev Psychiatry.

[10] Bryan CJ, Griffith JE, Pace BT, Hinkson K, Bryan AO, Clemans TA, Imel ZE (2015). Combat exposure and risk for suicidal thoughts and behaviors among military personnel and veterans: a systematic review and meta-analysis. Suicide Life Threat Behav.

[11] Sareen J, Houlahan T, Cox BJ, Asmundson GJG (2005). Anxiety disorders associated with suicidal ideation and suicide attempts in the National Comorbidity Survey. J Nerv Ment dis.

[12] Kessler RC, Borges G, Walters EE (1999). Prevalence of and risk factors for lifetime suicide attempts in the National Comorbidity Survey. Arch gen Psychiatry.

[13] Pirkis J, Burgess P, Dunt D (2000). Suicidal ideation and suicide attempts among Australian adults. Crisis.

[14] Oquendo MA, Galfalvy H, Russo S, Ellis SP, Grunebaum MF, Burke A, Mann JJ (2004). Prospective study of clinical predictors of suicidal acts after a major depressive episode in patients with major depressive disorder or bipolar disorder. Am J Psychiatr.

[15] Pfeiffer PN, Brandfon S, Garcia E, Duffy S, Ganoczy D, Myra Kim H, Valenstein M (2014). Predictors of suicidal ideation among depressed veterans and the interpersonal theory of suicide. J Affect Disord.

[16] Weissman MM, Klerman GL, Markowitz JS, Ouellette R (1989). Suicidal ideation and suicide attempts in panic disorder and attacks. N Engl J med.

[17] Sareen J, Cox B, Clara I, Asmundson G (2005). The relationship between anxiety disorders and physical disorders in the U.S. National Comorbidity Survey. Depression and Anxiety.

[18] Norton PJ, Temple SR, Pettit JW (2008). Suicidal ideation and anxiety disorders: elevated risk or artifact of comorbid depression?. J Behav Ther Exp Psychiatry.

[19] Seelig AD, Jacobson IG, Smith B, Hooper TI, Boyko EJ, Gackstetter GD, Gehrman P, Macera CA, Smith TC (2010). Millennium cohort study T: sleep patterns before, during, and after deployment to Iraq and Afghanistan. Sleep.

[20] Taylor KM, Hilton MS, Campbell SJ, Beckerly ES, Shobe K, Drummond PAS (2014). Prevalence and mental health correlates of sleep disruption among military members serving in a combat zone`. Mil med.

[21] Agargun MY, Kara H, Solmaz M (1997). Sleep disturbances and suicidal behavior in patients with major depression. The Journal of Clinical Psychiatry.

[22] Bernert RA, Joiner TE, Cukrowicz KC, Schmidt NB, Krakow B (2005). Suicidality and sleep disturbances. Sleep.

[23] Bernert RA, Joiner TE (2007). Sleep disturbances and suicide risk: a review of the literature. Neuropsychiatr dis Treat.

[24] Pigeon WR, Pinquart M, Conner K (2012). Meta-analysis of sleep disturbance and suicidal thoughts and behaviors. J Clin Psychiatry.

[25] Ribeiro JD, Pease JL, Gutierrez PM, Silva C, Bernert RA, Rudd MD, Joiner TE (2012). Sleep problems outperform depression and hopelessness as cross-sectional and longitudinal predictors of suicidal ideation and behavior in young adults in the military. J Affect Disord.

[26] Nadorff MR, Ellis TE, Allen GJ, Winer ES, Herra S (2014). Presence and persistence of sleep-related symptoms and suicidal ideation in psychiatric inpatients. Crisis.

[27] Malik S, Kanwar A, Sim LA, Prokop LJ, Wang Z, Benkhadra K, Murad MH (2014). The association between sleep disturbances and suicidal behaviors in patients with psychiatric diagnoses: a systematic review and meta-analysis. Syst rev.

[28] Chakravorty S, Grandner MA, Mavandai S, Perlis LM, Sturgis BE, Oslin WD (2014). Suicidal ideation in veterans misuising alcohol: Relationships with insomnia symptoms and sleep duration. Addict Behav.

[29] Bernert RA, Kim SJ, Iwata NG, Perlis ML (2015). Sleep disturbances as an evidence-based suicide risk factor. Curr Psychiatry rep.

[30] Bjorngaard JH, Bjerkeset O, Romundstad P, Gunnell D (2011). Sleeping problems and suicide in 75,000 Norwegian adults: a 20 year follow-up of the HUNT I study. Sleep.

[31] Tanielian T, Karney BR, Chandra A, Meadows SO (2014). The deployment life study: methodological overview and baseline sample description.

[32] Troxel WM, Shih RA, Pedersen E, Geyer L, Fisher MP, Griffin BA, Haas AC, Kurz J, Steinberg PS: Sleep in the Military: Promoting Healthy Sleep Among U.S. Servicemembers. Santa Monica, CA: RAND Corporation, 2015; 2015 10.7249/rhq-05-02-19 PMC5158299 28083395

[33] McCall WV, Black CG (2013). The link between suicide and insomnia: theoretical mechanisms. Curr Psychiatry rep.

[34] Roman V, Walstra I, Luiten PG (2005). Too litte sleep gradually desensitizes the serotonin IA receptor system. Sleep.

[35] American Psychiatric Association (1994). Diagnostic and Statistical Manual of mental disorders.

[36] American Psychiatric Association (2013). Diagnostic and Statistical Manual of mental disorders.

[37] Liu X (2004). Sleep and adolescent suicidal behavior. Sleep.

[38] Richardson JD, St Cyr KC, Nelson C, Elhai JD, Sareen J (2014). Sleep disturbances and suicidal ideation in a sample of treatment-seeking Canadian Forces members and veterans psychiatry research. Psychiatry Research.

[39] Cukrowicz KC, Otamendi A, Pinto JW, Bernert RA, Krakow B, Jointer TEJ (2006). The impact of insmonia and sleep disturbances on depression and suicidality. Dreaming.

[40] Nadorff MR, Nazem S, Fiske A (2011). Insomnia symptoms, nightmares, and suicidal ideation in a college sample. Sleep.

[41] Nadorff MR, Nazem S, Fiske A (2013). Insomnia symptoms, nightmares, and suicide risk: duration of sleep disturbance matters. Suicide Life Threat Behav.

[42] Pigeon WR, Britton PC, Ilgen MA, Chapman B, Conner KR (2012). Sleep disturbance preceding suicide among veterans. Am J Public Health.

[43] Hoffmire CA, Kemp JE, Bossarte RM: Changes in Suicide Mortality for Veterans and Nonveterans by Gender and History of VHA Service Use, 2000–2010. Psychiatric Services 2015, **0**(0):appi.ps.201400031. 10.1176/appi.ps.201400031 25930036

[44] Wittchen HU (1994). Reliability and validity studies of the WHO-Composite International diagnostic Interview (CIDI): a critical review. J Psychiatr res.

[45] Haro JM, Arbabzadeh-Bouchez S, Brugha TS, de Girolamo G, Guyer ME, Jin R, Lepine JP, Mazzi F, Reneses B, Vilagut G (2006). Concordance of the Composite International diagnostic Interview version 3.0 (CIDI 3.0) with standardized clinical assessments in the WHO World mental health surveys. Int J Methods Psychiatr res.

[46] Ratcliffe G, Enns M, Belik S, Sareen J (2008). Chronic pain conditions and suicidal ideation and suicide attempts: an epidemiologic perspective. Clinical Journal of Pain.

[47] Nelson C, Cyr KS, Corbett B, Hurley E, Gifford S, Elhai JD, Richardson JD (2011). Predictors of posttraumatic stress disorder, depression, and suicidal ideation among Canadian Forces personnel in a National Canadian Military Health Survey. J Psychiatr res.

[48] StataCorp.: Stata Statistical Software: Release 13**.** College Station, TX: StataCorp LP.; 2013.

[49] Ohayon MM (2002). Epidemiology of insomnia: what we know and what we still need to learn. Sleep med Reviews.

[50] Morin CM, Jarrin DC (2013). Epidemiogy of insomnia: prevalence, course, risk factors, and public health burden. Sleep med Clin.

[51] Hoge CW, Auchterlonie JL, Miliken CS (2006). Mental health problems, use of mental health services, and attrition from military service after returning from deployment to Iraq or Afghanistan. JAMA.

[52] Kessler RC, Chiu WT, Demler O, Walters EE (2005). Prevalence, severity, and comorbidty of 12-month DSM-IV disorders in the National Comorbidity Survey Replication. Arch gen Psychiatry.

[53] Pagura J, Fotti S, Katz LY (2009). Sareen J, the swampy Cree suicide prevention team: help seeking and perceived need for mental health care among individuals in Canada with suicidal behaviors. Psychiatr Serv.

[54] Carney CE, Segal ZV, Edinger JD, Krystal AD (2007). A comparison of rates of residual insomnia symptoms following pharmacotherapy or cognitive-behavioral therapy for major depressive disorder. J Clinical Psychiatry.

[55] Zayfert C, DeViva JC (2004). Residual insomnia following cognitive behavioral therapy for PTSD. J Trauma Stress.

[56] Long ME, Hammons ME, Davis JL, Frueh BC, Khan MM, Elhai JD, Teng EJ (2011). Imagery rescripting and exposure group treatment of posttraumatic nightmares in veterans with PTSD. J Anxiety Disord.

[57] Thunker J, Pietrowsky R (2012). Effectiveness of a manualized imagery rehearsal therapy for patients suffering from nightmare disorders with and without a comorbidity of depression or PTSD. Behav res Ther.

[58] Trockel M, Karlin B, Taylor C, Brown G, Manber R (2015). Effects of cognitive behavioral therapy for insomnia on suicidal ideation in veterans. Sleep.

[59] Miller LJ (2008). Prazosin for the treatment of posttraumatic stress disorder sleep disturbances. Pharmacotherapy.

[60] Raskind MA, Peskind ER, Hoff DJ, Hart KL, Holmes HA, Warren D, Shofer J, O'Connell J, Taylor F, Gross C (2007). A parallel group placebo controlled study of prazosin for trauma nightmares and sleep disturbance in combat veterans with post-traumatic stress disorder. Biol Psychiatry.

[61] Raskind MA, Peterson K, Williams T, Hoff DJ, Hart K, Holmes H, et al. A Trial of Prazosin for Combat Trauma PTSD With Nightmares in Active-Duty Soldiers Returned From Iraq and Afghanistan. Am J Psychiatry. 2013:170(9). 10.1176/appi.ajp.2013.12081133 23846759

[62] Krystal JH, Pietrzak RH, Rosenheck RA, Cramer JA, Vessicchio JC, Jones KM, et al. Sleep disturbance in chronic military-related PTSD: clinical impact and response to adjunctive Risperidone in the veterans affairs cooperative study #504. J Clin Psychiatry. 2016; 10.4088/JCP.14m09585 26890894

[63] Schoenfeld FB, Deviva JC, Manber R (2012). Treatment of sleep disturbances in posttraumatic stress disorder: a review. J Rehab res Develop.

[64] Bernardy NC, Friedman MJ (2015). Psychopharmacological strategies in the management of posttraumatic stress disorder (PTSD): what have we learned?. Curr Psychiatry rep.

[65] Pollack M, Hoge E, Worthington J, Moshier S, Wechsler R, Brandes M, Simon N (2011). Eszopiclone for the treatment of posttraumatic stress disorder and associated insomnia: a randomized, double-blind, placebo-controlled trial. J Clin Psychiatry.

[66] Ursano RJ, Kessler RC, Stein MB, Naifeh JA, Aliaga PA, Fullerton CS, Sampson NA, Kao TC, Colpe LJ, Schoenbaum M (2015). Suicide attempts in the US Army during the wars in Afghanistan and Iraq, 2004 to 2009. JAMA Psychiatry.

